# 

**Published:** 1849-07

**Authors:** 


PUBLISHED UNDER THE AUSPICES OF THE

**American Society of Dental Surgeons.**

EDITORS:

CHAPIN A. HARRIS, M. D., D. D. S.

AMOS WESTCOTT, M. D., D. D. S.

WILLIAM H. DWINELLE, M. D., D. D. S.

CORRESPONDENT FOR ENGLAND:

JAMES ROBINSON, D. D. S., 7 Gower-st. Bedford Square, London.

BALTIMORE:

ARMSTRONG & BERRY,

PHILADELPHIA: LINDSAY & BLAKISTON.

JOHN W. WOODS, PRINTER.

$5 00 per annum, payable in advance.

This Periodical weighs 9 ounces.

